# Targeting of Hepatic Macrophages by Therapeutic Nanoparticles

**DOI:** 10.3389/fimmu.2020.00218

**Published:** 2020-03-04

**Authors:** Clara I. Colino, José M. Lanao, Carmen Gutierrez-Millan

**Affiliations:** ^1^Area of Pharmacy and Pharmaceutical Technology, Department of Pharmaceutical Sciences, University of Salamanca, Salamanca, Spain; ^2^The Institute for Biomedical Research of Salamanca (IBSAL), Salamanca, Spain

**Keywords:** hepatic macrophages, nanoparticles, drug delivery, biodistribution, Kupffer cells, toxicity

## Abstract

Hepatic macrophage populations include different types of cells with plastic properties that can differentiate into diverse phenotypes to modulate their properties in response to different stimuli. They often regulate the activity of other cells and play an important role in many hepatic diseases. In response to those pathological situations, they are activated, releasing cytokines and chemokines; they may attract circulating monocytes and exert functions that can aggravate the symptoms or drive reparation processes. As a result, liver macrophages are potential therapeutic targets that can be oriented toward a variety of aims, with emergent nanotechnology platforms potentially offering new perspectives for macrophage vectorization. Macrophages play an essential role in the final destination of nanoparticles (NPs) in the organism, as they are involved in their uptake and trafficking *in vivo*. Different types of delivery nanosystems for macrophage recognition and targeting, such as liposomes, solid-lipid, polymeric, or metallic nanoparticles, have been developed. Passive targeting promotes the accumulation of the NPs in the liver due to their anatomical and physiological features. This process is modulated by NP characteristics such as size, charge, and surface modifications. Active targeting approaches with specific ligands may also be used to reach liver macrophages. In order to design new systems, the NP recognition mechanism of macrophages must be understood, taking into account that variations in local microenvironment may change the phenotype of macrophages in a way that will affect the uptake and toxicity of NPs. This kind of information may be applied to diseases where macrophages play a pathogenic role, such as metabolic disorders, infections, or cancer. The kinetics of nanoparticles strongly affects their therapeutic efficacy when administered *in vivo*. Release kinetics could predict the behavior of nanosystems targeting macrophages and be applied to improve their characteristics. PBPK models have been developed to characterize nanoparticle biodistribution in organs of the reticuloendothelial system (RES) such as liver or spleen. Another controversial issue is the possible toxicity of non-degradable nanoparticles, which in many cases accumulate in high percentages in macrophage clearance organs such as the liver, spleen, and kidney.

## Introduction

The use of particles as carriers of therapeutic agents for liver targeting is not a new idea. Hepatic nanoparticle uptake and distribution was initially studied as a drawback to be avoided because it entails a lack of specific selective distribution to other desired targets as well as toxicity and safety concerns. Nevertheless, from another point of view, strategies of selective delivery to different kinds of hepatic cells have also been explored in the search for specific targets of drugs included in nanoparticulated systems, such as hepatocytes, hepatic stellar cells, endothelial cells, and also the Kupffer cells (KC), the resident liver macrophages.

The liver is a very complex organ with many cells that are different in both morphology and functionality but which are nonetheless strongly inter-related. Although there are some other hepatic cells with phagocytic activity, KCs are undoubtedly the main ones responsible for phagocytosis in the liver. Moreover, it is estimated that they constitute 80–90% of all the macrophages present in the body (1).

Kupffer cells are the liver-resident macrophages, considered professional phagocytes to distinguish them from facultative ones. They are the largest mononuclear phagocyte population in the body and constitute approximately 20% of non-parenchymal liver cells. They present functional heterogeneity, likely due to their different origins and intrinsic plasticity (2).

They have an evident role in monitoring the blood entering the zone in order to endocytose debris, degenerated cells and any potentially harmful materials from the gut and circulation stream. Their strategic location at the luminal side of the hepatic sinusoidal endothelium allows them to act as sentinels that capture and process particles. They are involved in antigen presentation and processing and in the modulation of some hepatocyte functions.

Activation of Kupffer cells can induce a series of events to inhibit pathogen replication, recruit other immune cells into the liver, and activate them. On the other hand, interaction derived from infiltration immune cells leads to KC regulation (2). These complex and multiple inter-relationships with other hepatic cells and their concomitant role in immunological processes provide KCs with a wide variety of receptors that can be harnessed for their specific targeting (3, 4).

Due to the involvement of KCs in the evolution of many liver diseases, modulation of their activity may be used for therapeutic ends and nanosystems constitute promising alternatives for achieve this goal. In the present work, we will focus on the proposed nanosystems for targeting hepatic macrophages in order to improve pathological processes in the liver. The role of macrophages in liver diseases, the types of nanovehicles used, their characteristics, and the influence of the phenotype will be revised. Also, pharmacokinetic models for characterizing the biodistribution of NPs in the liver are addressed.

## Monocyte–Macrophage System (MPS)

Macrophages have been considered to be a part of different body systems throughout history. Previously considered as reticulo-endothelial system (RES) cells, nowadays, macrophages are generally considered to belong to the monocyte mononuclear phagocyte or monocyte macrophage system (MPS), originally defined as a cell lineage of promonocytes that give rise to monocytes that finally becomes macrophages in tissues. This concept was later reformulated to include dendritic cells (5), and recently also the concept of the MPS has been questioned, based on evidence that tissue resident macrophages may be a separate lineage seeded during embryonic development and capable of self-renewal. The newly proposed nomenclature classifies mononuclear phagocytes according to their ontogeny, location, and/or morphology (6). Regarding the phagocytic cells in liver, the main difference of the newly proposed nomenclature is that macrophages and derived monocyte cells are considered to be separate entities (7).

### Macrophages

Macrophages are specialized phagocytic cells strategically distributed in the body and specifically adapted to each tissue once they are settled. They are non-migratory cells that monitor their surrounding environment and process the material they engulf. They also recruit other immune cells, playing an important role in immune defense and homeostatic processes (8). To fulfill their functions, macrophages possess a wide range of sensing molecules specialized according to the tissue in which they are nested in each case.

Due to their central role in homeostasis, inflammation, and immunity, macrophages have arisen as interesting targets for therapeutic intervention (9).

Tissue macrophages are a very heterogeneous group of cells due to their different origins and their adaptation to the local environment. The contributions of embryonic origins and adult bone marrow cells vary depending on the tissues, with even tissue-resident macrophages of prenatal origin deriving from different hematopoietic stem cells.

**Figure 1:**
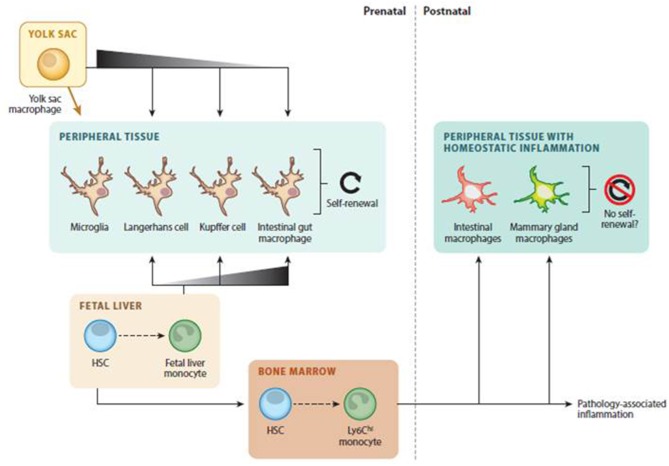
Tissue-resident macrophages development. Adapted with permission (9).

In view of the new evidence that questions the previous MPS model, it is postulated that there are two groups of macrophages in tissues: one coming from prenatally established populations and a second one originating from infiltrated monocytes that are more related to inflammatory conditions. Figure 1 illustrates the different origins and development processes of tissue-resident macrophages.

It has been demonstrated in mice that there are also several monocyte-derived tissue macrophage populations whose phenotypes reflect different origins. One such population is made up of macrophages originating from Ly-6C high expressing monocytes (classical monocytes), mainly coming from bone marrow, that express inflammatory chemokine receptors (like CCR2), pattern-recognition receptors, and cytokines. Another population is made up of macrophages derived from Ly-6C low expressing monocytes (non-classical monocytes), mainly coming from the spleen, that present a patrolling behavior and express more scavenging receptors. In the steady state, classical ones can leave the bloodstream and patrol extravascular tissues. They can be converted in some proportion into Ly-6C low expressing monocytes and transport antigens to lymph nodes. Non-classical monocytes patrol the intravascular spaces to clear dying endothelial cells. Under inflammation, classical monocytes differentiate to macrophages that are capable of self-renewal (10).

Despite the differences between mice and humans, genetic expression studies have demonstrated that these two subsets of monocytes are also present in humans, together with another intermediate subset between the classical and the non-classical (11, 12).

With respect to the macrophages that are present in the liver, although traditionally the term hepatic macrophages and Kupffer cells (KC) are used almost interchangeably, modifications to the MPS model mentioned above have also affected this assumption. After injury, heterogeneous hepatic macrophages populations can be observed, such as liver-resident macrophages or KCs and two subsets of bone marrow monocyte-derived macrophages (MoMFs), as well as peritoneal macrophages for subcapsular regions of the liver (13).

#### Classification

It is well-established in mice that macrophages can undergo two different activation states: M1 or classically activated and M2 or alternatively activated. M1 macrophages can produce pro-inflammatory cytokines and chemokines, high levels of reactive nitrogen and oxygen intermediates. They can also facilitate Th1 response and strong antimicrobial and antineoplastic effect. M2 ones are suppressive, involved in cellular repair and characterized by efficient phagocytic activity and high expression of scavenger, galactose, and mannose-type receptors. M1 and M2 even present different iron, glucose, and amino acid metabolism (2, 4). Although not much information is available regarding human beings, there is evidence to suggest a similar behavior in human macrophages (14). This traditional dichotomic classification seems to be too simplistic in view of recent increases in our knowledge of this area due to new sophisticated characterization techniques. Regarding Kupffer cells, their great flexibility and plasticity allow them to adopt a range of multiple intermediate phenotypes depending on the signals, which can lead to a broad spectrum of activation states (13, 15). Nevertheless, this simple classification into two possible extreme activation states is still used as a reference for KC behavior.

Differences not only in activation but also in origin have a great impact on the different subsets that can be defined for liver macrophages. The ontogeny and maintenance of resident hepatic macrophages have been the objects of many studies by important research groups, which have demonstrated that, besides the existence of self-renewal processes from prenatally settled local precursor cells, Kupffer cell populations are maintained by the infiltration of circulating bone-derived monocytes that differentiate into Kupffer cells in the liver (16, 17). Murine models have provided evidence that MoMF can contribute to regenerating the resident liver macrophage population when KC are massively depleted (18).

On the other hand, even some studies with models of sterile liver injury have shown phagocytes with an expression of the transcription factor GATA-6, suggesting macrophage infiltration from the peritoneal cavity (19).

## Role of Kupffer Cells in Hepatic Diseases: Cytokines and Chemokines

Due to their physiological functions, Kupffer cells are involved in local cell communication and homeostasis maintenance. The prominent role of KCs in immune processes, particle engulfment, antigen presentation, and the attraction and stimulation of T cells is well-known. They recruit other immune cells in the liver and release mediators to initiate response in other liver cells (2, 13).

Regarding other types of hepatic macrophages, it is known that bone marrow-derived macrophages participate in liver repair and regeneration but functional differences with Kupffer cells have not been clearly established. Following their activation, they produce cytokines that trigger a cascade of responses in other cells. If we accept the classical M1/M2 classification, it can be said that M1 KCs release proinflammatory cytokines, including tumor necrosis factor (TNF)-a, IL-6 and IL-1β, while M2 KCs release IL-4, IL-10, IL-13, and transforming growth factor-β. Consequently, the balance between these two phenotypes can ultimately lead to many different effects such as liver damage and wound repair (20).

The complex roles and expression of different KC phenotypes can lead to both protective and harmful responses (2, 21), and since it is required that their activation be precise, timely and localized, any KC dysregulation can lead to significant pathology (15). However, the results of some studies and hypotheses regarding these opposing effects are disputed. In some cases, KC depletion can be beneficial because of the reduction of inflammation or fibrosis but, on the other hand, the suppression of KC's role against pathogen invasion can clearly be harmful. Further studies must be done in order to conclude if KC depletion could prevent or exacerbate liver damage.

Thus, liver macrophages play an essential role in many pathogenic stages such as acute liver injury, fatty liver disease, fibrosis, cirrhosis, and liver tumors (22), constituting potential therapeutic targets for liver disease treatments. However, the pathogenesis of the disease treated and the phenotype of the macrophages targeted are points to consider when targeting macrophages (23).

In response to liver injuries such as alcoholic liver and non-alcoholic fatty liver disease (NAFLD) diseases, Kupffer cells are activated and a polarization to an M1-like phenotype is promoted in resident as well as monocyte-derived macrophages (24, 25). When injury ends, there is a switch to M2 restorative macrophages that release anti-inflammatory cytokines, regenerative growth factors, and matrix degrading metalloproteinase (MMP) expression, which promote tissue repair (26).

Macrophage polarization also has an important role in the growth and development of tumoral tissues. Macrophages in tumor tissues (TAMs) are mostly M2-like cells that produce tumor-promoting cytokines and growth factors that promote tumor expansion, angiogenesis, metastasis, and immune cell evasion. TAMs also contribute to drug resistance (27, 28).

## Targeting Liver Macrophages With NPs

### Delivery Systems for Macrophages Recognition and Targeting

Liver macrophages are specialized in the internalization of foreign nanoparticles, playing an essential role in its destination in the organism, since they are involved in their uptake and trafficking *in vivo*. Therapeutic capacity and clearance mechanisms in clinically relevant nanomedicines have been linked to macrophage activity. However, due to their pathophysiological roles in diseases (29), liver macrophages are also potential therapeutic targets for a variety of aims, from cell activation to monocyte recruitment or macrophage differentiation (30). Emerging nanotechnology systems may offer new perspectives for macrophage vectorization. After intravenous administration, nanoparticles are opsonized in the bloodstream before being phagocytized by macrophages and accumulated in the RES organs. This passive targeting promotes the accumulation of the NPs in the liver, a process that increases within tumors due to the EPR (enhanced permeability and retention) effect (31).

Kupffer cells internalize NPs through multiple scavenger, toll-like, mannose, and Fc receptors (23). The mechanisms involved are macropinocytosis, clathrin-mediated endocytosis, caveolin-mediated endocytosis, and additional endocytotic pathways (1, 32, 33). Clathrin-mediated endocytosis has been pointed out as responsible for the internalization of size ranges of approximately 100–350 nm, while caveolin-mediated mechanism is responsible for the endocytosis of 20–100 nm particles (34–36). Macropynocitosis allows for large volume extracellular internalization of 0.5–5 μm nanosystems (23). The internalization process is then modulated by the size of the NPs as well as other characteristics such as charge. Larger nanoparticles generally show more efficient hepatic uptake: a diameter >200 nm is preferred for liver deposition (1, 37–39). Charged NPs, especially those with a positive charge, are taken up to a greater extent than those with a neutral charge (23, 39). Shape also has a great impact on NP uptake and elongated NPs are taken up less by macrophages than spherical ones (40). However, cylindrical silica NPs showed the highest accumulation in the liver compared to other shapes (41). Moreover, surface hydrophobic NPs tend to be opsonized by proteins that make them attractive to the phagocytic cells of the MPS (42–44).

Therefore, the success of therapeutic strategies targeting liver macrophages involves the use of different NP types with appropriate properties to allow them to be preferentially taken up by the liver. Incorporation of ligands to the surface of NPs increases specificity through active targeting (21, 31).

#### Liposomes

Liposomes are biodegradable vesicles with an aqueous core and a phospholipidic membrane that can carry hydrophilic as well as hydrophobic compounds. They have the advantage of being both biocompatible and biodegradable, whereas their instability is one of their main drawbacks. Macrophages, especially KCs, readily phagocytose circulating liposomes (45), causing them to accumulate in the liver (46). The liposomes proposed for the vectorization to hepatic macrophages have mainly sizes of ~100 nm due to restrictions on parenteral formulations. This passive targeting has been exploited for the administration of anti-infective drugs that have an effect on liver macrophages, some examples of which are shown in Table 1. For instance, commercial liposomal formulations of amphotericin B (Ambisome) allow the drug to accumulate in spleen and liver macrophages that constitute a reservoir of *Leishmania*, reducing the drug's nephrotoxicity (54, 55). Besides, the encapsulation of vancomycin in liposomes improves its poor penetration into cells, allowing its targeting to Kupffer cells. In a mouse model, such formulations reduced the intracellular Methicillin-resistant *Staphylococcus aureus* (MRSA) reservoir where the bacteria can survive and proliferate, significantly increasing the survival rate of infected mice over those treated with the drug solution (48, 49).

**Table 1:** Characteristics of some liposomes proposed for liver macrophages targeting.

**Composition**	**Size/nm**	**Active**	**Effect**	**References**
HSPC, CHOL, DSPG	80	Amphotericin B	Leishmanicide	(47)
DCP, DMPG, CHOL	527.6 ± 58.2	Vancomycin	Improvement of MRSA infection	(48, 49)
DPPC: PEG-(2000)-DSPE: NBD-PE:CHOL	100	Dexamethasone	Switch to M2 phenotype Liver injury and liver fibrosis reduction	(50)
EPC:CHOL	100–150	Curcumin 1,25-dihydroxy-vitamin D3 (calcitriol)	Switch to M2 phenotype Reduction in liver inflammation, fibrosis and fat accumulation	(51)
DOPC: DOPE	83.5–108.8	Arginin-like ligands	Switch to M1 phenotype Antitumor	(52)
DSPC: CHOL: Mannose	~95	Muramyl dipeptide (MDP)	Increase of Kupffer cells tumoricidal activity	(53)

*HSPC, hydrogenated soy phosphatidylcholine; CHOL, cholesterol; DSPG, distearoyl phosphatidylglycerol; DSPC, distearoyl 3 phosphatidylcholine; DCP, dicethylphosphate; DMPG, dimyristoylphoshatidylglycerol; DPPC, dipalmitoyl phosphatidylcholine; PEG-(2000)-DSPE, polyethyleneglycol-(2000)-distearoyl phosphatidylethanolamine; NBD-PE, N-(7-Nitrobenz-2-oxa-1,3-diazol-4-yl)-1,2-dihexadecanoyl-sn-glycero-3-phosphoethanolamine, triethyl-ammonium salt); EPC, egg phosphatidylcholine; DOPC, 1,2-dioleoyl-sn-glycero-3-phosphocholine; DOPE, 1,2-dioleoyl-sn-glycero-3-phosphoethanolamine*.

Liposomes are also suitable vehicles for the targeting of anti-inflammatory compounds such as dexamethasone, curcumin, or calcitriol to the liver. They show improved results over the free drug in the treatment of acute and chronic liver disease models in mice. Pharmacokinetic studies show the preferential uptake of the liposomes by the liver although they accumulate not only in Kupffer cells but also in monocytes, infiltrating macrophages and, to a lesser extent, T cells. Liposomes also induce a repolarization of macrophages to a regulatory phenotype (50, 51).

Pathological conditions may influence the liposomes behavior, and recently, changes in spatial distribution and a decrease in the liposomes uptake by macrophages has been described in liver fibrosis (56).

Liposomes can be decorated for active vectorization with surface modifiers, such as mannose that has been proposed for the treatment of liver tumors in order to increase liposome uptake by macrophages via receptor-mediated endocytosis. The increase in mannose ligand concentration leads to a higher accumulation percentage in the livers of mice. Also, active targeting of an immunomodulator to liver by mannose-decorated liposomes resulted in more effective inhibition of metastasis than when delivered by liposomes without mannose (53, 57).

Substances attached to liposome surface may produce other effects. Some arginine-like ligands may switch macrophages to the M1 phenotype in order to achieve an antitumor effect. In a study with a library of this kind of ligands, nitroarginine, and acetylglutamine DOPE:DOPC liposomes were the most effective for the redirection of macrophage phenotypes (52).

In summary, preferentially uptake of liposomes by liver macrophages make them suitable vehicles for the vectorization of anti-inflammatory, anti-infective or other drugs for the treatment of liver diseases. When associated with anti-inflammatory compounds, they are able to promote the macrophages' regulatory state and reduce the dose for the treatment of acute and chronic liver injury. The decoration of the surface of liposomes with arginine-like and mannose ligands may be useful for anti-tumor treatments.

#### Lipoplexes

Inhibition of regulatory pathways triggered by macrophages via the use of gene therapies is another strategy for the treatment of macrophage-associated diseases. Lipid-based NPs are the most successful non-viral vehicles for targeting RNAi to Kupffer cells and can reach a high efficiency of transfection. Although they are sometimes referred to as liposomes, lipoplexes are usually different in both structure and composition. They are based on cationic lipids that are able to both bind and condense negatively charged iRNA through electrostatic interactions and to deliver the payload into the cytoplasm of target cells (58). They also incorporate neutral lipids in order to attenuate the toxicity of cationic lipids. Besides cell selectivity, the success of these kinds of therapies depends on transfection efficacy. Lipoplexes are engulfed by Kupffer cells through macropinocytosis and clathrin-mediated mechanisms after IV administration, but RNAi escape from endosomes is a rate-limiting step for these therapies (59). Proper lipid design allows RNAi to reach the cellular cytoplasm where it exerts its action. In this way, cationic lipid C12-200 eposide would prevent RNAi lysosomal degradation (58) through a micropinocytosis internalization mechanism as macropinosomes do not follow the endosomic degradation pathway (23). It was applied to inhibit the PD-1/PD-L1 pathway that contributes to the persistence of viral liver infections. Lipid NPs of 70–80 nm size were efficiently internalized (66.5%) and expressed by Kupffer cells after *in vivo* IV administration in viral-infected mice. This led to an enhanced antiviral effect. This promising antiviral immunotherapy may be applicable to vaccine development, to treat diverse viral liver infections and other diseases such as hepatocarcinome (60).

However, cationic lipids are used as vehicles for RNAi forming lipoplexes. A high transfection efficacy in Kupffer cells can be achieved with proper lipid selection, showing promising results in immunotherapy.

#### Inorganic Nanoparticles

Inorganic NPs are a broad group of metallic and non-metallic nanomaterials. Some of them are non-biodegradable, which constitutes a pitfall for their use. However, they possess excellent properties such as small size, high surface area, and easy functionalization and may induce *per se* responses in macrophages with different therapeutic applications as shown in Table 2.

**Table 2:** Some examples of inorganic nanoparticles for liver macrophage targeting.

**NPs type**	**Size/nm**	**Model**	**Effect**	**References**
CeO_2_	53.36 ± 7.04	Lipopolysaccharide induced severe sepsis in rats	Reduced expression of inflammatory macrophage mediators	(61)
Au	7.4 ± 1.6	Rat liver injury with ethanol and methamphetamine	Downregulation of Kupffer cells activity	(62)
Glucomannan-silica	27.6 ± 0.6 −28.89 ± 1.60	Murine inflammatory bowel disease	M2 polarization	(63)
SPIONs				
Dimercaptosuccinic acid	65	Murine and human M2 cells	Modification of M2 activation profile	(64)
3-Aminopropyl-triethoxysilane	54			
Aminodextran	150			

Inorganic NPs have been used in the diagnostic and treatment of liver fibrosis and recently this topic has been reviewed in depth (65). For instance, the reduction of inflammatory macrophage activity caused by ceriumoxide NPs has been proposed to prevent hepatic dysfunction in septic rats. The NPs attenuate the expression of a number of different inflammatory macrophage mediators that are associated with sepsis, improving rat survival (61). This downregulation of Kupffer cell activity was also reported for gold nanoparticles (GNPs) in two rat liver-injury models causing antioxidant and antifibrotic effects (62).

Inflammatory diseases may also be treated by the switch of macrophages from an inflammatory (“M1”) to an anti-inflammatory (“M2”) phenotype. Carbohydrates are able to induce phenotypic changes promoting one or other activation state depending on their physical and chemical characteristics (66). As an example, glucomannan carbohydrate-decorated silicon oxide nanoparticles promote M2 polarization in macrophages by inducing clustering of mannose receptors (MR) on the cell surface. Although this was assayed in a murine inflammatory bowel disease model, it may be applied to other inflammatory diseases (63).

Conversely, induction of an immune response was the aim of calcium phosphate polyetilenimine/SiO_2_ nanoparticles used as carriers of a Toll-like-3 ligand. The NPs targeted the liver with 30–40% NP-positive cells when administered intravenously to mice and could be applied to vaccination (67).

Another group of inorganic NPs with applications in liver macrophage vectorization is superparamagnetic iron oxide nanoparticles (SPIONs), which are promising nanomaterials as diagnostic, iron supplement, and drug carrier agents. Surface modifications can render a high biocompatibility (64). They are phagocytized by macrophages and induce a pro-inflammatory response (68–70) through the activation of the Toll-like receptor 4 (71). Recently, they have been proposed for the reeducation of M2 tumor-associated macrophages to an antitumor M1 state in cancer treatment. This effect was studied for carboxymethyldextran-coated iron oxide NPs (Ferumoxytol®), which are approved by the FDA for the treatment of iron deficiency and other clinical uses. In a mouse *in vivo* model, these NPs inhibited tumor growth and prevented metastasis development. This activity was associated with the increase of M1 macrophages that may have been promoted by iron overload (72, 73). The uptake mechanism of carboxy-dextran coated SPIONs by human macrophages is a clathrin-mediated and scavenger receptor endocytosis although macropinocytosis may also contribute to internalization (74). The recognition of SPIONs by macrophages depends on the particle size and surface modifier, and it is better for positively charged particles and for a size of ~60 nm of size, although such SPIONs show cytotoxicity (71, 74, 75).

The efficient SPION uptake by macrophages allows its use for labeling macrophages in a cellular therapy for the treatment of liver cirrhosis. The labeled cells were tracked *in vivo* by magnetic resonance and no effects on phagocytic activity or cell viability were observed (75). AuNPs have also been used to this end and 50 nm was proposed as the optimal size for labeling without toxicity concerns for both NPs types (76).

In summary, inorganic nanoparticles are able to stimulate and also inhibit the activity of macrophages. Moreover, they can promote a switch to a specific macrophage state. The desired effect depends on the disease to be treated. The interaction of SPIONs with macrophages has been well-characterized and a mathematical model for the prediction of NPs uptake has even been developed (74). However, further insights are needed to clarify the relationship between the characteristics of NPs and their therapeutic and toxic effects.

#### Polymeric NPs

Polymeric NPs are colloid systems made of natural or synthetic polymers. They consist of a matrix in which the drug is homogeneously distributed or may be structured in a nucleus and a polymeric shell (nanocapsules) (54). They display great versatility. A fundamental feature of these nanosystems is their biodegradability, which is essential for intravenous administration.

**Figure 2:**
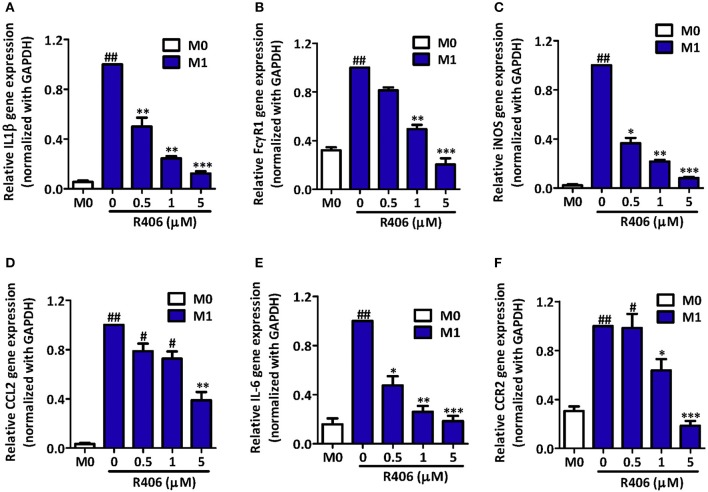
Inhibition of M1-specific differentiation and inflammatory markers by R406 in RAW macrophages. Gene expression of M1 markers IL-1β **(A)**; FcγR1 **(B)**; iNOS **(C)**; CCL2 **(D)**; IL-6 **(E)**; and CCR2 **(F)** in RAW 264.7 cells after incubation with medium alone (M0) or M1 stimulus with R406 (0, 0.5, 1, and 5 μM). Expression values for the respective genes in untreated M1 macrophages were set at 1.0 to calculate the relative gene expression. Data are presented as mean + SEM. #*p* < 0.05, ##*p* < 0.01 denotes significance versus control M0 macrophages. *p < 0.05, **p < 0.01, and ***p < 0.001 denotes significance versus M1-differentiated macrophages. Reproduced with permission (78).

Polymers of polylactic–glycolic acids (PLGA) are the most used for drug delivery as they are compatible and biodegradable compounds, and are excipients approved by the FDA. NPs of these polyesthers show excellent properties as drug carriers for liver macrophages vectorization (77) in order to improve efficacy or reduce side effects. PLGA NPs have been proposed as carriers of an inhibitor of the spleen tyrosine kinase SYK. This enzyme is overexpressed in M1 macrophages and shows a positive correlation with the pathogenesis of NASH and alcoholic hepatitis in patients. Although the bare inhibitor was more effective *in vitro*, its incorporation to 160 nm PLGA nanoparticles improved its intrahepatic delivery and therapeutic efficacy *in vivo*. This ameliorated fibrosis, inflammation, and steatosis in mice after IV administration (Figure 2) (78).

The objective pursued for rosiglitazone NP incorporation was to reduce their serious side effects such as the increased risk of fatal cardiac arrhythmia. Rosiglitazone vectorization to macrophages using 200 nm PLGA/polyvinylic acid (PVA) nanospheres allows for its selective delivery to circulating monocytes and Kupffer cells, reducing obesity-related inflammatory reaction in the white adipose tissue and liver and mitigating undesired effects (79).

Cationic polymers have been proposed as carriers of genetic material to reduce the expression of proteins related to liver diseases. This was the objective of RNAi for protein NOGOB encapsulation in [poly(amine-co-ester) (PACE) terpolymers] NPs. NOGOB promotes M1 polarization, stimulating the progression of alcoholic liver disease and liver fibrosis. The *in vivo* spleen administration of the NPs with a size of 240–300 nm allowed up to 60% Nogo-B protein suppression (80). This is a high transfection efficacy although alternative administration routes adequate for clinical use should be assayed. High transfection efficacy was also achieved by PLAcore/PVAshell NPs loaded with PEI-CD98 siRNA. The objective was the downregulation of CD98, a factor that is overexpressed in the livers of non alcoholic fatty liver disease (NAFLD) patients playing as a key inducer in this disease. The IV administration of CD98siRNA NPs with a size of 273.1 ± 19.3 nm effectively targeted liver hepatocytes and Kupffer cells, leading to a significant decrease of major proinflammatory cytokines and markers of NAFLD (81).

Chitosan, a biodegradable, positively charged polymer of natural origin is also widely used as a vehicle of RNAi. Quaternary chitosan NPs were designed for vectorization to macrophages of the RNAi of proinflammatory cytokine tumor necrosis factor TNFα. The NPs with sizes between 210 and 279 nm and zeta potentials from 14 to 22 mV achieved a high cellular uptake efficiency near 100% by RAW-274-7 macrophages. NPs cross-linking with TPP reduced their size and zeta potential, resulting in better transfection abilities (82). Polyethylenimine (PEI) is another cationic polymer with high transfection efficacy due to the proton–sponge phenomenon. In order to increase the chitosan's ability to transfect and reduce the PEI cytotoxicity, both components are mixed. NPs of sizes from 150 to 200 nm with both polymers achieved high macrophages transfection *in vitro* with non-cell toxicity (83). Functionalization of chitosan and other polymers, such as dendrimers with mannose, allows for active vectorization with a better selectivity for macrophage vectorization of RNAi and drugs (84–86).

The applications of polymeric NPs extend to HIV infections, where macrophages play a central role as virus reservoirs. Kutscher et al. designed GLU-decorated chitosan (CS) shell and polylactic-co-glycolic acid (PLGA) core nanoparticles (GLU-CS-PLGA) that recognize special receptors expressed in infected macrophages for the delivery of the antiretroviral drug nevirapine (87). Also, polymeric NPs modified with folic acid may target atazanavir/ritonavir to activated macrophages that overexpress folate receptor at an elevated level (88).

Polyesthers and chitosan are the polymers most often used in order to target drugs and RNAi to liver macrophages. To this end, NPs sizes of 150 to 300 nm have been prepared. Decoration with mannose and other ligands allow NPs to be selectively uptaken.

#### Other Nanosystems

Exosomes are phospholipidic nanoparticles of endosomal origin that are secreted by cells. They display similar advantages to synthetic nanoparticles, but they usually show higher biocompatibility and physiological activity. Like the majority of nano-sized vesicles, they also accumulate in the liver and may target Kupffer cells. Exosomes derived from mesenchymal stem cells reduce the levels of proinflammatory factors in murine macrophages *in vitro* with a decrease in biochemical and histological damage (89). These effects were also observed in an *in vivo* experimental lethal hepatic injury mouse model. The beneficial effect of these vesicles on reducing mortality implies modulation of the inflammatory response and activation of protective mechanisms to limit cell death (90).

Mesenchymal stem cell exosomes are then new types of nanovehicles with anti-inflammatory and protective effect in liver injury that constitute promising strategies for liver disease treatments.

## Impact of Macrophage Phenotype in NPs Uptake and Toxicity

Nanosystems that specifically target and deliver therapeutics to polarized macrophages are of interest due to the role that they play in liver diseases. In order to improve the design of those drug delivery systems, the interaction of nanoparticles with macrophages of different phenotypes must be understood, which is why it has been the subject of several studies. Increased NP sequestration by M1 phenotypes has been reported as they are involved in biological processing of foreign materials. Thus, incorporation of phagocytosis promoters in lipid-latex nanoparticles allowed them to target inflammatory M1 macrophages (91). Also, a higher sequestration by inflammatory phenotype was found *in vitro* and *in vivo* for spherical silica nanoparticles. This was attributed to the silanol terminal groups that would attach to the receptors of anionic groups that are overexpressed in M1 macrophages (92).

However, M2 macrophages show increased expression of mannose and galactose receptors (93). This makes it possible to target specifically anti-inflammatory phenotypes with mannose–decorated nanoparticles (94). Pluronic and chitosan-based NPs of 150 to 265 nm, decorated with mannose, with positive and negative zeta potentials, respectively, can selectively target M2 macrophages to treat inflammatory diseases and HIV infections. The charge of the NPs and their degree of internalization are dependent on mannose density (84, 94).

Recently, a comparative study on gold nanoparticle uptake by human monocyte-derived macrophages of different phenotypic polarization showed, in general, higher internalization of gold nanoparticles by M2-polarized human macrophages in comparison with the M1-polarized cells. The extent of the uptake was positively correlated with the expression of M2 markers CD163 and CD206. Further investigation in human Kupffer cells showed comparable internalization of nanoparticles by those unstimulated Kupffer cells with a mixed M1/M2 phenotype and the M2-polarized cells, both of which ingested more nanoparticles than the M1-polarized cells did (28).

In short, various types of nanosystems have been designed for targeting polarized macrophages, although further research is necessary to define the NP characteristics necessary for promoting preferential M1 or M2 uptake.

## Hepatic Biodistribution of Nanoparticles

The kinetics of nanoparticles when administered *in vivo* strongly affects their therapeutic efficacy. Nanoparticular systems are recognized by macrophages of the mononuclear phagocyte system and tend to accumulate mainly in organs such as the liver, the spleen, or the lungs (95). The selective distribution of nanoparticular systems in these types of organs facilitates their use for the diagnosis and treatment of different types of pathologies.

Experimental studies conducted with cell lines and animals have brought about a clarification of the mechanisms of penetration into macrophages and hepatocytes using chitosan nanoparticles (96).

**Figure 3:**
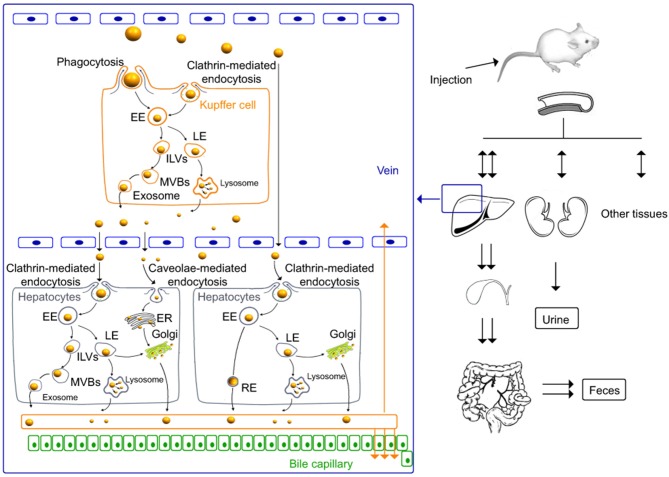
*In vivo* distribution and elimination of chitosan nanoparticles in Kupffer cells and rat hepatocytes (96). International Journal of Nanomedicine. Reproduced with permission from Dove Medical Press Ltd.

Figure 3 shows the arrangement of chitosan nanoparticles in Kupffer cells, hepatocytes, and in whole animals.

As shown in Figure 3, *in vivo* studies in mice demonstrate that, at the level of Kupffer cells, the cellular uptake of chitosan nanoparticles is produced via mechanisms of phagocytosis and clathrin- and caveolin-mediated endocytosis and their release through the lysosomal and multivesicular pathways. In addition, nanoparticles penetrate intracellularly into hepatocytes. The renal and hepatobiliary excretion pathways constitute the main routes of elimination *in vivo*, observing a slow elimination with a nanoparticle half-life of >60 days (96). In addition, *in vitro* studies conducted with murine macrophage cell lines show that chitosan nanoparticle uptake was clathrin-mediated endocytosis as a primary mechanism and also via phagocytosis as a secondary mechanism. After internalization, a large proportion of the nanoparticles may be excreted from the cells by lysosome-mediated and multivesicular body-mediated exocytosis (97).

Short-term biodistribution of silica nanoparticles in mice demonstrates their high accumulation in organs of the reticulo-endothelial system such as liver and spleen. At the same time, the animals in the experiment showed a clear increase in the number of hepatic macrophages over time. Aggregates of macrophages or microgranulomes increased between 6 and 24 h after silica nanoparticle administration. Clearance of silica nanoparticles from the liver appears to be slower than from the spleen, probably due to hepatic processing and biliary excretion (98).

Other authors also describe the retention of silica nanoparticles and the development of fibrosis in rat livers up to 60 days after IV administration (99).

Iron oxide nanoparticles are a type of system increasingly used for magnetic resonance imaging in diagnostic techniques (100). The administration of polyacrylic acid-coated iron oxide nanoparticles is associated with a selective distribution in the liver that produces proinflammatory activation and liver toxicity in mice. A high accumulation of iron was also observed by macrophages phagocytosis in the periportal zone of the hepatic acinus of the liver and in the splenic red pulp of the spleen which demonstrates the specific uptake of this type of nanoparticles by the monocyte–macrophage system (101).

Nanosystems are currently being combined with cell-based platforms with interesting biodistribution properties and with different therapeutic objectives.

As an example, plasmonic gold nanostars (GNS) were incorporated into immune system cells such as dendritic cells or macrophages obtained from bone marrow in order to investigate the biodistribution of these types of cells in a murine lymphoma model (102).

**Figure 4:**
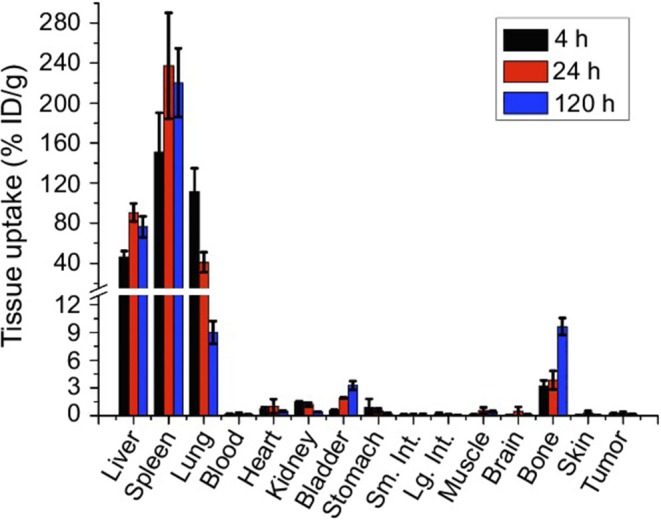
Biodistribution of GNS-labeled macrophage cells at different times after IV administration in mice (102). International Journal of Nanomedicine. Reproduced with permission from Dove Medical Press Ltd.

Figure 4 shows biodistribution in organs and tissues at different times in mice after IV administration of GNS-labeled macrophage cells. The quantification of gold in the different tissues was performed using an ICP-MS technique (102).

As shown in Figure 4, after IV administration of GNS-labeled macrophage cells, a specific distribution of this cell-based nanosystem preferably in the spleen, liver, and lung was observed, indicating that these organs had high macrophage cell accumulation. This type of cell-based delivery system presents interesting applications for cell-tracking studies (102).

Stabilin (-1 and -2) are specific receptors for the cellular uptake of different substances such as antisense oligonucleotides in the liver through clathrin-mediated endocytosis (103). Recent studies have been conducted to determine the mechanisms involved in the uptake of nanoparticles by hepatic macrophages. For this reason, an embryonic zebrafish model has been used to evaluate the interaction of nanoparticles with macrophages and endothelial cells using liposomes as nanoparticles. This research has demonstrated the role of the stabilin-2 receptor in the uptake of nanoparticles by endothelial cells. The nanoparticle uptake proved to be independent of the type of material and the functional properties of the nanoparticles but was influenced by the surface charge of the nanoparticle. In addition, the interaction between endothelial cells and nanoparticles can be blocked by competitive inhibitors of stabilin-2 such as dextran sulfate (104).

In another work about the mechanisms involved in the anti-inflammatory activity and in the recognition of crystals and nanomaterials by macrophages, cell-surface receptors or membrane cholesterol have been described as mechanisms involved in crystal nanoparticle recognition although other phagocytosis mechanisms are still unknown (105).

Pathological conditions may also influence NPs uptake. Liver fibrosis profoundly changes the myeloid compartment in the liver, with decreasing numbers of Kupffer cells and increasing numbers of MoMF. With the aim of investigating the changes in the targeting properties of different nanosystems in hepatic fibrosis, Ergen et al. studied the biodistribution of three intravenously injected carrier material, i.e., 10 nm poly(N-(2-hydroxypropyl)methacrylamide) polymers, 100-nm PEGylated liposomes, and 2,000 nm poly(butyl cyanoacrylate) microbubbles, in two fibrosis mice models. They found a decreased uptake of polymers and microbubbles by almost all myeloid cells of the fibrotic liver. However, liposomes had an overall higher targeting efficiency for endothelial and myeloid cells, which remained high even in fibrotic livers with around 60% carrier positive cells in healthy livers and after induction of liver fibrosis, although with a low specificity for the various cell populations. In all cases, Kupffer cells and monocyte-derived macrophages were the cells with increased percentage of carrier-positive cells (56).

### Pharmacokinetic Models

Different pharmacokinetic models such as compartmental and especially physiologically based pharmacokinetic (PBPK) models have been developed to characterize the disposition of drugs in different organs and tissues, and especially in the liver, when they are administered in different types of nanoparticles (98, 106–112).

**Figure 5:**
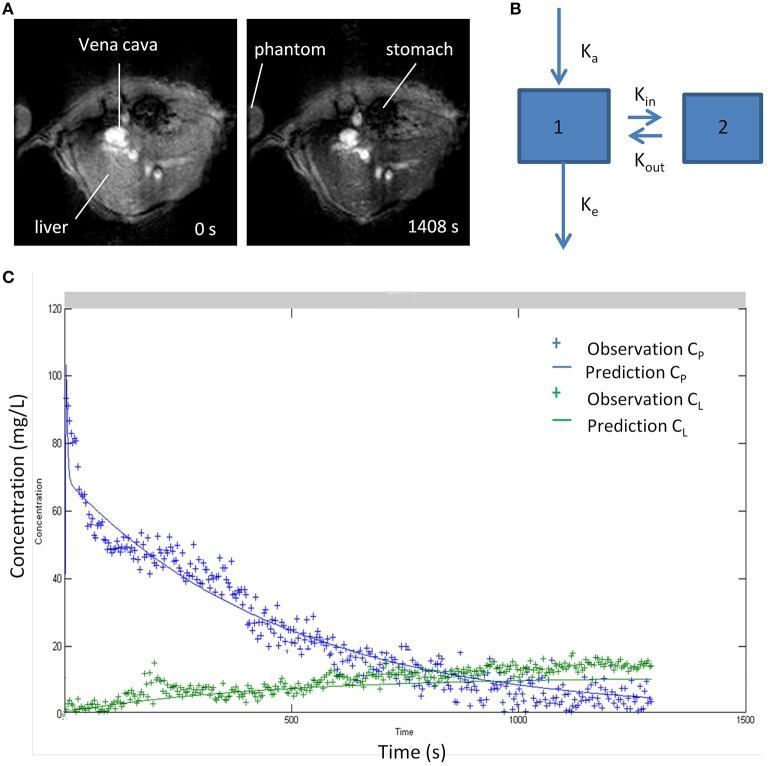
**(A)** MR images of liver before and after 20 min of intravenous injection of iron oxide nanoparticles **(B)** Two-compartment kinetic model used to characterize blood pharmacokinetics and distribution in liver tissue of superparamagnetic iron oxide nanoparticle (SPIO). Compartments 1 and 2 represent blood and liver. **(C)** Pharmacokinetic profiles of nanoparticles in blood and liver fitted to the two-compartment model (106). Reproduced with permission.

Classic pharmacokinetic models, such as the two-compartmental model, have been proposed to characterize the accumulation in the reticulo-endothelial system and in the livers of mice, as well as the elimination of superparamagnetic iron oxide nanoparticles (SPIONs). The model allows binding to Kupffer cells and extrahepatic clearance of nanoparticles to be characterized using dynamic magnetic resonance imaging (MRI) as seen in Figure 5 (106). The constants *K*_in_ and *K*_out_ describe the kinetics of nanoparticles associated with and dissociated from the macrophage, and the constant *K*_e_ describes the elimination of nanoparticles from the blood compartment by the extrahepatic RES (97).

The kinetic parameters of distribution in the liver *K*_in_ and *K*_out_ are related to Kupffer cell numbers, allowing the function of the reticulo-endothelial system to be evaluated in different situations and presents therapeutic applications in liver disease, allowing the chronic liver injury to be evaluated, taking into account that the macrophages are integrated in different stages of the inflammatory process (106).

### Physiologically Based Pharmacokinetic (PBPK) Models

PBPK models have been developed to characterize nanoparticle biodistribution in organs of the reticuloendothelial system (RES) such as the liver or the spleen.

PBPK models constitute an interesting strategy for modeling and simulation that allow the kinetic behavior of drugs in animals and humans to be predicted with a physiological basis. These models are based on grouping the body into different compartments that are assimilated to different organs and tissues. These compartments are defined by the volume of tissue and the blood flow that irrigates it. The mass balance of the drug throughout the body is defined through first-order differential equations. The distribution of the drug in each of the tissues can be perfusion-limited or diffusion-limited (112–114). Perfusion rate-limited kinetics occurs when blood flow limits tissue distribution. In the steady state, the concentration of drug in the tissue is in balance with the concentration of drug in the blood through a specific partition coefficient for each tissue. This type of distribution occurs in lipid molecules that easily penetrate the tissue. Permeability rate-limited kinetics occurs when permeability across the membrane constitutes the limiting process of intratisular distribution and occurs mostly in more polar or hydrophilic molecules (112, 113).

**Figure 6:**
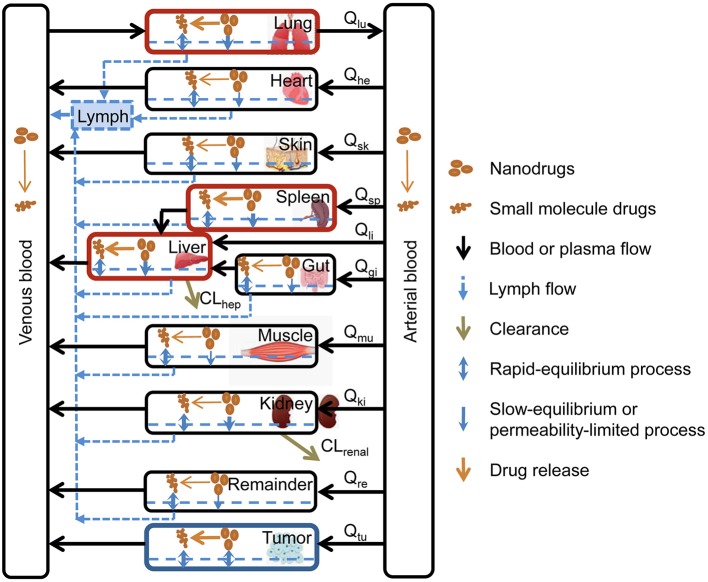
Specific PBPK model to characterize the biodistribution of nanoparticles (112). Reproduced with permission.

Specific PBPK models that consider the specific disposition properties of nanoparticles have been developed to characterize the biodistribution and elimination of nanoparticulate systems in the whole body. This model assumes the existence of two PBPK submodels: one to characterize the disposition of the nanoparticles and another to characterize the disposition of the free drug as shown in Figure 6. This model predicts a greater accumulation of nanoparticles in the liver, spleen, and lungs due to the vascular structure of these tissues and the uptake of nanoparticles by the mononuclear phagocytic system. On the other hand, this type of model has some limitations, given that certain processes, such as aggregation or degradation of nanoparticles, among others, can change the properties of nanoparticles and their arrangement in different organs and tissue. In addition, this type of model allows the interaction between the drug, the nanoparticle, and the complex physiology of the organism to be modeled and simulated (112).

Some of these PBPK models have been designed to characterize the role of macrophages in drug tissue concentrations by combining the data from clinical studies with *in vitro* data. The model allows the concentration of the antibiotic moxifloxacin in tissues from biopsies to be predicted, including those from interstitial fluid, intracellular fluid, vascular space, and macrophages. The study showed that macrophages contribute to the accumulation of the drug in tissues from biopsies (109).

This same type of PBPK model has also been used to predict behavior in liver diseases such as liver cirrhosis. This model allows changes in the plasma concentrations of different drugs, such as alfentanil or lidocaine among others associated with Child-Pugh class A, B, and C liver cirrhosis, to be predicted (115).

Previously, general PBPK models that allow the distribution of drugs incorporated in different types of nanoparticles to be simulated have been described (111). The model was tested with different kinds of nanoparticles with differences in drug dose, size, charge, shape, or surface properties. This model also considers saturable phagocytosis of the nanoparticles. This model is based on another previously published model to characterize the biodistribution of PAA-PEG (116).

This model considers 10 anatomical compartments and each compartment is divided into three subcompartments that represent blood, tissue, and phagocytic cells.

Bigger nanoparticles are better recognized by the macrophages than smaller ones. On the other hand, cationic nanoparticles are better recognized by macrophages than anionic or neutral ones (112, 117). Based on this model, when nanoparticles are injected into the blood, they are captured by phagocytic cells of organs of the reticulo-endothelial system (RES) such as the liver and the spleen depending on their electric charge, size, and agglomeration state, among other factors (111).

In the field of toxicokinetics, PBPK models have been applied to characterize the biodistribution of silver nanoparticles compared to ionic silver. The PBPK model predicts a higher accumulation of silver in the liver as silver nanoparticles in comparison with ionic silver (118).

## Nanoparticle Toxicity

The toxicity of nanosystems is an important issue that may limit its applicability. Once in the organism, the processing and final fate of nanoparticles is dependent on their composition.

Liposomes are biodegradable phospholipidic-based nanosystems, and once administered, they are degraded by serum proteins in the blood circulation or by intracellular lipases. The degradation products of liposomes are their constituent lipid molecules that can be further metabolized by the body. This also holds for other lipids forming solid lipid nanoparticles. However, positively charged lipids show limited compatibility as they may induce cytokine activation and cellular toxicity with apoptosis (119).

Polymeric nanoparticles are modified in the organism, giving rise to constituent monomeric units or modified polymer chains. For biodegradable polymers, degradation products are smaller than the renal molecular weight cutoff size and can follow renal elimination (120, 121). For non-biodegradable components, the larger molecules may be cleared by hepatobiliary or the mononuclear phagocyte system, but their biotransformation in hepatocytes and macrophages may cause toxicity (122, 123). As their lipidic counterparts, cationic polymers such as PEI show high cellular toxicity (1).

The non-biodegradability of some inorganic NPs is a factor that leads to liver toxicity (124) and limits their applicability in humans. When NPs are not decomposed by the phagocytosis process, they will remain within the cell and be sequestered in the spleen and liver for long periods of time (122, 125–127). Once the nanoparticle-filled phagocyte dies, those nanoparticles are taken up again by other phagocytes of the same organ, resulting in a similar total amount of nanoparticles accumulated (128). NPs accumulate in the liver and especially in Kupffer cells (129) and could cause fibrosis and other histological tissue changes. They produce oxidative stress that in turn modulates the autophagic process in the liver, disrupting liver metabolism and homeostasis (65). This hepatic oxidative damage has been reported in both *in vitro* and *in vivo* models for AuNPs, SiO_2_NPs, and AgNPs, with differences among the NP compositions (130–134). However, long-term effects need to be further characterized (65).

Tunable properties of NPs, such as shape, surface charge, and size, may affect their toxicity (135, 136). For instance, cerium oxide (CeO_2_) NPs with rod-like shape showed higher and dose-dependently enhanced macrophage cytotoxicity responses with respect to cubic/octahedral NPs (137). The surface charge is also an important parameter that affects NP clearance rate. One study with mesoporous silica NPs (MSNs) showed that positively charged NPs were rapidly excreted from the liver into the gastrointestinal tract while negatively charged NPs remained sequestered in the liver (138). Also, the degradation of silica NPs to silicic acid for their renal excretion depends on the characteristics of the NPs, such as porosity, size, and surface chemistry (139–141).

Toxicity also depends greatly on the dose of the NPs, with cytotoxicity reported over certain doses for inorganic nanoparticles such as cerium oxide and gold NPs (61, 142, 143).

Therefore, tailoring the characteristics of NPs and controlling the doses of inorganic NPs used could overcome the toxicity problems in the liver and allow for their clinical application (65).

Biodegradable SPIONs or SiNPs offer a safer alternative. For instance, SPION administration is well-tolerated, and long-term *in vivo* biodistribution studies have shown that they can be transformed to non-superparamagnetic iron forms and eliminated with no signs of toxicity (64). However, in another study, toxicity of SPIONs and ultrasmall superparamagnetic iron oxide nanoparticles (USPIO) in human macrophages has been described (144).

## Conclusions

Different types of NPs such as liposomes, solid lipid nanoparticles, inorganic NPs, or exosomes have been proposed for targeting liver macrophages in order to treat liver diseases. They are used as delivery systems but may also induce changes in macrophage phenotypes with influence in the progression of the illness. Lipids and polymeric NPs as vectors of RNAi may exert therapeutic effects by inhibiting regulatory pathways triggered by macrophages. Moreover, the interaction of NPs with macrophages depends on their phenotype, although this issue must be studied in more depth. The physiological-based pharmacokinetic models have been mainly used to describe and simulate NPs destination in the organism. Although strategies to vectorize liver macrophages with NPs are promising, compatibility issues, especially long-term toxicity, are drawbacks for certain systems, especially non-biodegradable ones.

## Author Contributions

All authors have equally contributed to the manuscript conception and preparation. JL and CC elaborated an abstract and an index and then all the three authors organized a well-balanced distribution of the different items. For their part of the work, the responsible person looked for the references, redacted the text and selected the images. CG-M was responsible of references database organization. CG-M and CC adapted the format to the journal template. JL, CG-M, and CC revised the whole content and approved the submitted version.

### Conflict of Interest

The authors declare that the research was conducted in the absence of any commercial or financial relationships that could be construed as a potential conflict of interest.
